# Estimation of the risk of secondary malignancy arising from whole-breast irradiation: comparison of five radiotherapy modalities, including TomoHDA

**DOI:** 10.18632/oncotarget.8392

**Published:** 2016-03-26

**Authors:** Eun Young Han, Nava Paudel, Jiwon Sung, Myonggeun Yoon, Weon Kuu Chung, Dong Wook Kim

**Affiliations:** ^1^ Department of Radiation Physics, The University of Texas MD Anderson Cancer Center, Houston, TX, USA; ^2^ Department of Radiation Oncology, University of Arkansas for Medical Sciences, Little Rock, AR, USA; ^3^ Department of Bio-Convergence Engineering, Korea University, Seoul, Korea; ^4^ Department of Radiation Oncology, Kyung Hee University Hospital at Gangdong, Seoul, Korea

**Keywords:** secondary malignancy, breast, IMRT, VMAT, TomoHDA

## Abstract

The risk of secondary cancer from radiation treatment remains a concern for long-term breast cancer survivors, especially those treated with radiation at the age younger than 45 years. Treatment modalities optimally maximize the dose delivery to the tumor while minimizing radiation doses to neighboring organs, which can lead to secondary cancers. A new TomoTherapy treatment machine, TomoHDA^TM^, can treat an entire breast with two static but intensity-modulated beams in a slice-by-slice fashion. This feature could reduce scattered and leakage radiation doses. We compared the plan quality and lifetime attributable risk (LAR) of a second malignancy among five treatment modalities: three-dimensional conformal radiation therapy, field-in-field forward-planned intensity-modulated radiation therapy, inverse-planned intensity-modulated radiation therapy (IMRT), volumetric modulated arc therapy, and TomoDirect mode on the TomoHDA system. Ten breast cancer patients were selected for retrospective analysis. Organ equivalent doses, plan characteristics, and LARs were compared. Out-of-field organ doses were measured with radio-photoluminescence glass dosimeters. Although the IMRT plan provided overall better plan quality, including the lowest probability of pneumonitis, it caused the second highest LAR. The TomoTherapy plan provided plan quality comparable to the IMRT plan and posed the lowest total LAR to neighboring organs. Therefore, it can be a better treatment modality for younger patients who have a longer life expectancy.

## INTRODUCTION

Evolving early-stage breast cancer treatment strategies have improved the survival of patients who undergo breast conservation surgery. After surgery, patients generally receive radiation treatment of the entire breast with a dose of 50.4 Gy [1]. This strategy reduces local cancer recurrence by one half to two thirds and the chance of death due to breast cancer by about one sixth [2].

However, among >10-year survivors, the probability of developing a secondary malignancy increases significantly in women who receive the radiation treatment at the age of 45 years or younger [3]. To improve these patients' long-term survival and quality of life, minimizing scattered and leakage radiation dose to normal tissues or organs while maintaining tumor control becomes more critical [4].

Various treatment modalities have been applied for breast cancer treatment using a traditional linear accelerator (Linac). Patients can be treated by three-dimensional conformal radiation therapy (3D-CRT), field-in-field forward-planned intensity-modulated radiation therapy (FinF), standard intensity-modulated radiation therapy (IMRT), and volumetric modulated arc therapy (VMAT). The newly introduced TomoHDA^TM^ machine (v2.0, Accuray, Madison, WI, USA) can deliver IMRT beams at static angles in a slice-by-slice fashion while a patient slowly moves into the beam. This treatment modality is called TomoDirect^TM^ (TOMO). Another advanced feature of the TomoHDA system is that two field-defining jaws move dynamically to conform to a target in the craniocaudal direction, which can produce a sharp dose fall-off.

Previous comparative studies have shown that the risk of secondary cancer induction is higher with an IMRT plan than with a 3D-CRT plan. This is attributed to increased out-of-field leakage radiation due to the higher number of fields and monitor units (MUs) used in the IMRT plan. But the plan quality of the IMRT plan, with regard to factors such as planning target volume (PTV) dose coverage and organ-at-risk (OAR) dose reductions, is generally better than that of the 3D-CRT plan [5-9]. In a comparative study of oropharyngeal cancer treatment plans, Gestel et al. reported that the helical TomoTherapy plan elicited better homogeneous PTV dose coverage and better OAR sparing than other modalities. However, the lifetime attributable risks (LARs) between treatment modalities were not compared [10]. The purpose of the study described here is to compare the plan quality and LAR among five treatment modalities (3D-CRT, FinF, IMRT, VMAT, and TOMO). Our hypothesis is that the TomoDirect modality (TOMO) can maintain the plan quality of the Linac-based IMRT plan and still lower LAR compared with the other treatment modalities.

## MATERIALS AND METHODS

### Patient selection and planning parameters

Ten female patients with breast cancer (5 left sided and 5 right sided) were retrospectively selected for this study. As shown in Table 1, PTVs among the ten patients ranged from 291 to 1421 cm^3^.

**Table 1:** Patient characteristics

Patient number	Breast site	Tangential beam angles (o)	PTV volume (cm^3^)
1	Left	317.4 / 127.2	1421
2	Left	323.6 / 129	733
3	Left	305.9 / 118.8	596
4	Left	319.7 / 127.8	291
5	Left	315.7 / 122.2	726
6	Right	239.3 / 49.1	485
7	Right	235.0 / 38.1	1025
8	Right	233.5 / 35	866
9	Right	243.6 / 50	904
10	Right	233.1 / 41	381

A CT simulator (Brilliance CT, Philips Medical System, Netherlands) was used to obtain planning CT images with 3 mm slice thickness for patients in supine position with both arms up with an aid of a wing board. TOMO plans for all patients were optimized with the TomoTherapy (v 5.0) planning system, and the Linac-based (21iX Varian Medical System, Palo Alto, CA) plans were optimized with the Eclipse treatment planning system (v 8.9, Varian Medical System, Palo Alto, CA).

PTV was defined as the “ipsilateral” breast (the breast containing the tumor) with 5 mm skin extraction. The lungs, heart, and contralateral breast were contoured as OARs. The prescription dose was set to 50.4 Gy in 28 fractions, and the plan was normalized as this dose covering 95% of the PTV. Dose constraints for ipsilateral lung volumes receiving 20 Gy and 10 Gy doses (V20_Gy_ and V10_Gy_) were set as less than 20% and 40% of the lung volume, respectively. The maximum spinal cord dose was limited to less than 45 Gy [11]. Doses to the other organs were kept as low as possible.

**Figure 1:**
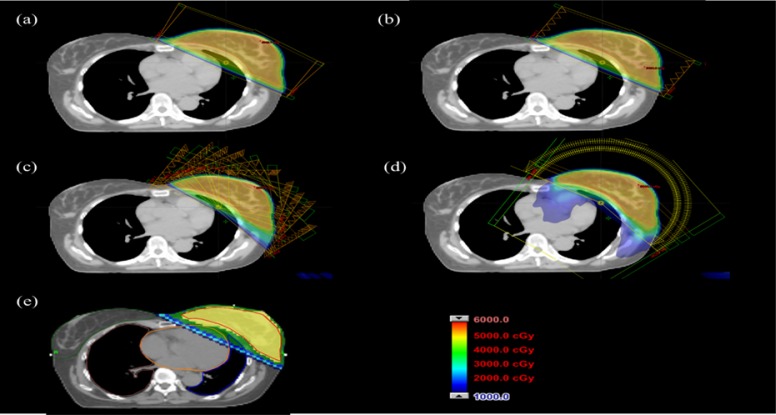
Graphical view of the isodose lines in the axial view for the patient 1 corresponding to Table 1 for the **a.** three-dimensional conformal radiation therapy (3D-CRT) plan, **b.** field-in-field forward-planned intensity-modulated radiation (FinF) plan, **c.** intensity-modulated radiation therapy (IMRT) plan, **d.** volumetric modulated arc therapy (VMAT) plan, and **e.** TOMO plan.

For each patient, five different plans were created using the five treatment modalities. Figure 1 shows a graphical view of dose distributions and beam arrangements of the treatment plans. The 3D-CRT plan consisted two parallel opposed tangential beams. The FinF plan has the same gantry angles as the 3D-CRT plan but added sub-fields created by a multi-leaf collimator (MLC) for dose compensation. The IMRT plan consisted as IMRT field can consist any number of beams ∼>5 of 10 to 12 fields to spare the contralateral breast, lung, and heart. Recently, Li et al. reported a non-isocentric IMRT treatment strategy for breast radiation therapy [12], with significant reduction of ipsilateral lung and heart doses; however, this technique takes longer planning and treatment time, instead we generated a single isocentric IMRT plan and increased the number of fields to ensure PTV dose coverage. The range of the gantry angles of the IMRT plans was relatively larger than that of the 3D-CRT plan by ∼ 26.5^o^ ± 9.8^o^. The VMAT plan consisted of 3 to 4 partial arcs covering a range of beam angles similar to that of the IMRT plan. For the left breast irradiation, the average gantry angle spanned from 305^o^ to 152^o^, and for the right breast irradiation the average gantry angle spanned from 60^o^ to 214^o^. For the TOMO plan, a field width of 5.0 cm, a pitch of 0.4, and a maximum modulation factor of 2.067 were used.

In this study, contralateral breast, contralateral and ipsilateral lung, and “contralateral” and “ipsilateral” heart (corresponding to right-sided and left-sided breast cancers, respectively) were considered in-field organs. Six out-of-field organs—thyroid, stomach, liver, colon, gonad, and rectum—were selected and analyzed. Doses to in-field organs were derived from dose-volume histograms (DVHs), and doses to out-of-field organs were derived from measurements using a radio-photoluminescence glass dosimeter (RPLGD).

### Out-of-field organ dose measurement using a radio-photoluminescence glass dosimeter

**Figure 2:**
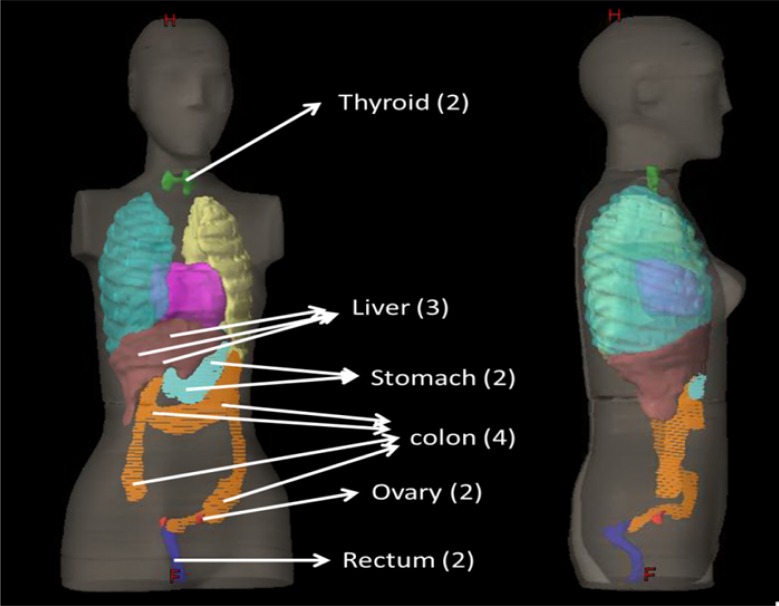
Detector positions in out-of-field organs (with numbers of RPLGDs in parentheses) in the female RANDO phantom

Out-of-field dosimetry requires a direct measurement as commercial treatment planning systems cannot estimate dose outside the radiation field well [13, 14]. We used RPLGD dosimeters (A.T.G., Chiyoda Technology Corporation, Tokyo, Japan) to estimate the out-of-field organ doses. RPLGD has good reproducibility and low energy dependency at energy levels > 200 keV [15, 16]. Small differences in individual sensitivity, repeatable readout, and excellent accuracy and stability at room temperature make RPLGD suitable for the dosimetry of scattered radiation outside the treatment field [17, 18]. Rah et al. reported that the reproducibility, fading effect, and angular dependency of RPLGD with a 6 MV photon beam were 0.9%, 1.7%, and 1.0%, respectively [19, 20]. All patient plans were delivered on an anthropomorphic RANDO female phantom (The Phantom Laboratory, Salem, NY) including two breasts (10.8 cm in diameter and 4.3 cm in height) attached to a bottom plate, and organ doses were collected with detectors placed in the approximate middle of each organ. A total dose of 10 Gy was delivered due to limited sensitivity of RPLGD to scattered radiation. The average dose measured by the detectors was considered the organ equivalent dose (OED). Figure 2 displays detector positions in out-of-field organs in the female RANDO phantom, including the numbers of RPLGDs in parentheses.

### In-field organ doses based on dose-volume histograms

Dosimetry for in-field organs (ipsilateral and contralateral lung, ipsilateral or contralateral heart, and contralateral breast) was based on the DVHs of each plan. The homogeneity index (HI), coverage index (CVI), and conformity index (CI) of the PTV and doses to lungs and heart were compared by treatment modality [21]. HI represents dose uniformity, defined as *HI* = (|*D2* - *D98*|/*Rx) × 100*, where *D2* and *D98* are the doses received by 2% and 98%, respectively, of the PTV in the DVH and Rx is the prescription dose [22]. By definition, smaller values of HI give better dose homogeneity in the PTV. CVI is defined as *CVI = V100/V_PTV_*, where *V100* is a volume receiving 100% of the prescription dose, and *V_PTV_* is a volume of PTV [22]. CI is defined as *CI = (V95_PTV_ × V95_PTV_)/(V_PTV_ × V95)*, where *V95_PTV_* is the PTV volume receiving 95% of the prescription dose, and *V95* is the body volume receiving 95% of the prescription dose [23]. In addition, the ipsilateral lung volumes receiving 20 Gy (V20_Gy_) and 10 Gy (V10_Gy_) are considered important parameters for evaluating the probability of pneumonitis [24-28].

We used the plateau dose-response model to estimate an OED for normal organs as follows [29, 30].

$$OED=\frac{1}{V}\sum_{i}V_{i}\left(\frac{1-\exp\left(-\delta D_{i}\right)}{\delta }\right),$$ (1)

where *V* is a body volume, and *V_i_* is a volume element exposed to a dose element *D_i_* [31]. In this model, a parameter *δ* was used to determine a dose-response curve for a specific organ. The OED values were used to estimate the LAR to each organ.

### Estimation of lifetime attributable risk to radiation dose

As in the BEIR VII: Health Risks from Exposure to Low Levels of Ionizing Radiation report [32], the LAR for a person exposed to a radiation dose (*D*) at age (*e*) is expressed as follows:

$$LAR\left(D,e\right)=\int_{e+L}^{100}{\left(ERR\left(D,e,a\right)\cdot \lambda_{I}^{C}\right)}^{w}\times {\left(EAR\left(D,e,a\right)\right)}^{1-w}\frac{s\left(a\right)}{s\left(e\right)},$$ (2)

where *ERR* and *EAR* are an excess relative risk and an excess absolute risk, respectively, attained at the age of *a* as a result of the radiation exposure at the age of λ^C^_I_ i is the baseline cancer incidence rate and *w* is the weight. S(*a*)/S(*e*) is a ratio of the probability of surviving at the ages of *a* and *e,* and *L* is the latent period (5 years) for solid tumors. A weight (*w)* of 0.7 is encouraged by the BEIR Committee for most organs, although exceptions include breasts and lungs. For breasts, only the EAR model is recommended, and for lungs, a weight of 0.3 is recommended. The EAR and ERR model of BEIR VII are functions of sex (*x*), age at exposure (*e*), and attained age (*a*) as defined by the following equation:

$$LAR\left(D,e\right)=\int_{e+L}^{100}{\left(ERR\left(D,e,a\right)\cdot \lambda_{I}^{C}\right)}^{w}\times {\left(EAR\left(D,e,a\right)\right)}^{1-w}\frac{s\left(a\right)}{s\left(e\right)},$$ (3)

where *β_s_*, γ, and η are model parameters for the age of exposure (*e*) and the attained age (*a*). Table 2 shows the parameters for preferred risk models in the BEIR VII report. In this study, we summed the age from *e + L* to 100 years in LAR calculations for consistency with data from International Commission on Radiological Protection (ICRP) Report 103 [33].

**Table 2:** Parameters for preferred risk models in the BEIR VII: Health Risks from Exposure to Low Levels of Ionizing Radiation report

Site	ERR model				EAR Model			
	β_M_	β_F_	γ	η	β_M_	β_F_	γ	η
Lung	0.32	1.4	−0.3	−1.4	2.3	3.4	−0.41	5.2
Breast (a<50)	Not used					9.4	−0.51	3.5
Breast (a>=50))						9.4	−0.51	1.1
Thyroid	0.53	1.05	−0.83	0	Not used			
Liver	0.32	0.32	−0.3	−1.4	2.2	1	−0.41	4.1
Stomach	0.21	0.48	−0.3	−1.4	4.9	4.9	−0.41	2.8
Colon	0.63	0.43	−0.3	−1.4	3.2	1.6	−0.41	2.8
Gonad	0.27	0.45	−0.3	−2.8	6.2	4.8	−0.41	2.8
Rectum	0.63	0.43	−0.3	−1.4	3.2	1.6	−0.41	2.8

## RESULTS AND DISCUSSION

### Comparison of the organ equivalent doses of the five treatment modalities

Plan statistics for our 10 patients generated using the five treatment modalities are tabulated in Table 3. While the TOMO plans produced better dose homogeneity in the PTV, the IMRT and VMAT plans produced better PTV dose coverage and dose conformity. The V20Gy of the ipsilateral lung was the lowest in the IMRT plan, followed by the VMAT, 3D-CRT, TOMO, and FinF plans. However, The V10Gy was the highest for the VMAT plan among the five modalities. Therefore the IMRT plan produced the least probability of radiation-induced pneumonitis.

**Table 3:** Patient plan information and findings (mean ± standard deviation) for the five modalities

Region	Site	Item	3D-CRT	FinF	IMRT	VMAT	TOMO
	No. of fields/arcs		2	2	10 to 12	3 to 4	2 to 4
	Monitor units per fraction		229 ± 13	220 ± 11	1479 ± 271	460 ± 51	2807 ± 655
In field	PTV coverage	HI	13.02 ± 1.78	11.74 ± 2.29	10.62 ± 2.18	11.41 ± 1.83	6.67 ± 2.81
		CVI	1.44 ± 0.18	1.41 ± 0.25	1.13 ± 0.06	1.10 ± 0.08	1.34 ± 0.16
		CI	0.60 ± 0.09	0.62 ± 0.10	0.82 ± 0.03	0.83 ± 0.04	0.51 ± 0.08
	Ipsilateral lung	V10^a^	16.88 ± 5.65	17.50 ± 6.82	11.66 ± 4.73	29.32 ± 10.02	17.18 ± 6.19
		V20^a^	12.93 ± 5.15	13.48 ± 6.27	6.97 ± 3.59	8.98 ± 5.17	13.42 ± 5.68
	Ipsilateral lung	OED^b^	2.42 ± 0.44	2.49 ± 0.43	2.33 ± 0.64	4.69 ± 0.87	2.26 ± 0.40
	Contralateral lung	OED	0.21 ± 0.12	0.20 ± 0.10	0.20 ± 0.10	1.23 ± 1.05	0.25 ± 0.07
	Ipsilateral heart	OED	1.77 ± 0.62	1.86 ± 0.63	1.42 ± 0.52	4.77 ± 0.75	1.99 ± 0.65
	Contralateral heart	OED	0.78 ± 0.58	0.69 ± 0.21	0.50 ± 0.08	2.59 ± 1.11	0.62 ± 0.13
	Contralateral breast	OED	0.35 ± 0.13	0.38 ± 0.14	0.29 ± 0.18	1.31 ± 0.55	0.31 ± 0.07
Out of field	Thyroid	OED	0.38 ± 0.13	0.37 ± 0.14	0.65 ± 0.21	0.76 ± 0.43	0.19 ± 0.04
	Liver	OED	0.45 ± 0.17	0.47 ± 0.16	0.65 ± 0.13	0.86 ± 0.39	0.36 ± 0.10
	Stomach	OED	0.45 ± 0.21	0.48 ± 0.20	0.65 ± 0.13	0.72 ± 0.18	0.25 ± 0.07
	Colon	OED	0.14 ± 0.03	0.16 ± 0.09	0.49 ± 0.13	0.30 ± 0.10	0.15 ± 0.02
	Gonad	OED	0.03 ± 0.01	0.04 ± 0.01	0.15 ± 0.05	0.10 ± 0.02	0.06 ± 0.01
	Rectum	OED	0.02 ± 0.01	0.02 ± 0.01	0.10 ± 0.03	0.06 ± 0.01	0.05 ± 0.01

a Percentage of treatment volume that received 10 Gy or 20 Gy.

b Organ equivalent dose (in Gy).

**Figure 3:**
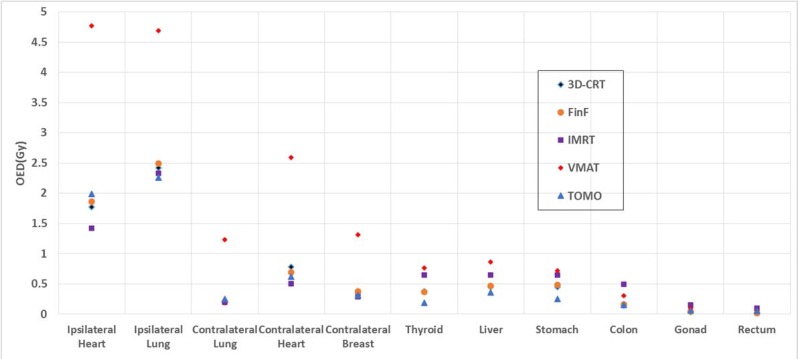
OEDs of in-field and out-of-field organs for the five treatment modalities

As shown in Figure 3, the OEDs in in-field organs were the highest with the VMAT plans. Most in-field OEDs were the lowest with the IMRT plans, but the out-of-field OEDs for IMRT were higher than those for the 3D-CRT, FinF, and TOMO plans. The average number of MUs of the IMRT plans was 7 times that of the 3D-CRT plans. This large MU value was caused by the high modulation of MLCs. This high modulation with IMRT possibly caused the higher dose for out-of-field OARs such as the colon, gonad, and rectum as shown as Table 3.

**Figure 4:**
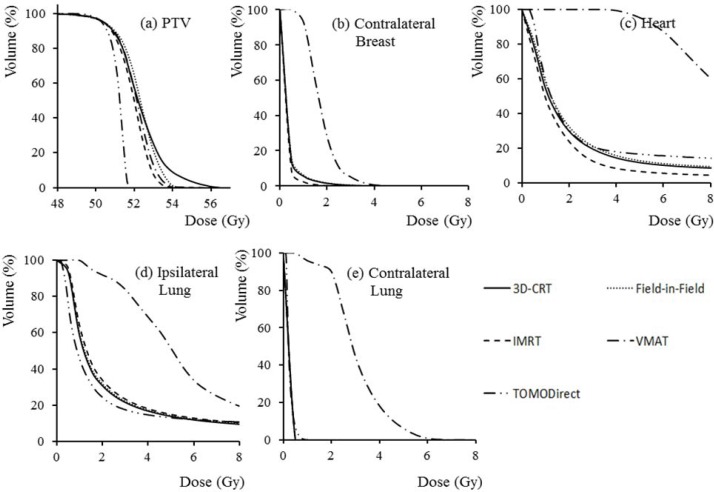
The dose-volume histograms for the **a.** planning target volume, **b.** contralateral breast, **c.** heart, **d.** ipsilateral lung, and **e.** contralateral lung of one patient for the five treatment modalities.

Even though the VMAT plan used lower MUs as compared to the IMRT plan, the VMAT plans resulted in higher OEDs to the majority of the critical structures due to the higher volume of irradiation. This tendency is similar to the results of Lee et al. [6]. We found that the TOMO plans offered the lowest OEDs in most of the out-of-field organs, while its in-field OEDs were comparable to those of the IMRT plan. Figure 4 shows an example of the DVHs for the PTV, contralateral breast, heart, ipsilateral lung, and contralateral lung of a representative patient. These results show that the plan quality of the TOMO plans was better or comparable to that of the IMRT plans.

### Comparison of lifetime attributable risk of the five treatment modalities

**Figure 5:**
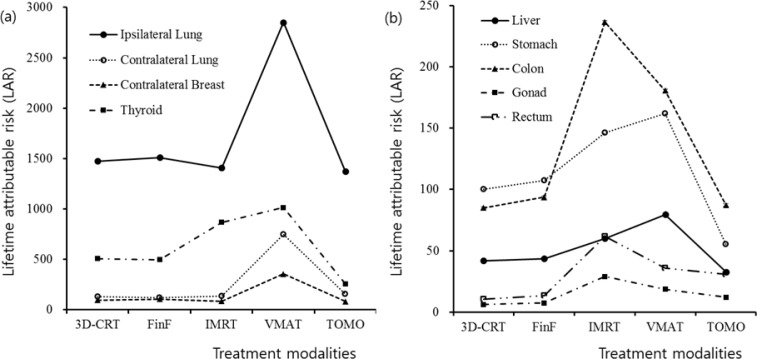
The lifetime attributable risk of secondary malignancy in each organ at risk for the five treatment modalities **a.** Ipsilateral lung, contralateral lung, contralateral breast, and thyroid. **b.** Liver, stomach, colon, gonad, and rectum.

Table 4 shows for each modality the estimated LAR (based on calculations or measurements) to the organs of a secondary malignancy for an exposure at age between 30 and 100 years. As shown in Figure 5, LAR for each organ depends on its distance from the primary beam and the used modality. Therefore, higher LARs were obtained in the ipsilateral lung, thyroid, contralateral lung, and contralateral breast, in that order. In in-field organs, the LARs were the highest for the VMAT plans, followed by the IMRT plans. The higher LARs in the out-of-field organs for the IMRT and VMAT plans are attributed to large MUs and larger volume of irradiation [34]. The values of LAR of the TOMO plans were comparable to or lower than those of the 3D-CRT and FinF plans. For the LAR of all of the organs totaled, the TOMO plans had a value of 2083 ± 255 among 100,000 persons, which is the lowest value compared with the other modalities. The LAR of all organs for the VMAT plan was twice that of the TOMO plans. Therefore, the VMAT plans had especially high risk relative to the others.

**Table 4:** The lifetime attributable risk (LAR) of secondary malignancy in the organs at exposure summed to 100 years (mean ± standard deviation)

Site	Lifetime attributable risk (among 100,000 population)				
	3D-CRT	FinF	IMRT	VMAT	TOMO
Ipsilateral lung	1474 ± 270	1511 ± 263	1408 ± 390	2851 ± 532	1374 ± 244
Contralateral lung	129 ± 73	120 ± 61	134 ± 53	746 ± 639	154 ± 40
Contralateral breast	95 ± 34	104 ± 38	83 ± 46	355 ± 148	83 ± 19
Thyroid	506 ± 176	496 ± 192	868 ± 280	1013 ± 573	255 ± 55
Liver	42 ± 16	44 ± 15	60 ± 12	79 ± 36	33 ± 10
Stomach	100 ± 47	107 ± 45	146 ± 29	162 ± 40	55 ± 15
Colon	85 ± 51	94 ± 55	237 ± 80	181 ± 60	87 ± 12
Gonad	6 ± 3	7 ± 3	29 ± 10	19 ± 4	12 ± 1
Rectum	11 ± 4	14 ± 4	62 ± 21	36 ± 5	30 ± 5
Total	2448 ± 340	2497 ± 341	2965 ± 493	5442 ± 1024	2083 ± 255

An interesting finding was that the TOMO plans produced the lowest maximum and the highest mean dose (95% of the prescription dose) to the skin of the ipsilateral breast, while the IMRT plans provided the maximum skin dose (Table 5). This advantage for patients treated with TOMO can eliminate the use of a bolus to maintain good dose coverage while minimizing adverse effects on skin.

**Table 5:** Skin doses (Gy) of five treatment modalities

	3D-CRT	FinF	IMRT	VMAT	TOMO
Minimum Dose	18.5 ± 6.4	19.6 ± 4.4	11.1 ± 3.9	11.3 ± 2.8	23.6 ± 7.7
Maximum Dose	56.3 ± 1.3	55.0 ± 1.0	57.2 ± 1.1	54.6 ± 1.5	53.4 ± 1.1
Mean Dose	43.7 ± 1.9	43.2 ± 1.4	41.6 ± 2.4	39.4 ± 2.3	47.9 ± 1.1

Some studies have reported that uncertainty in out-of-field dose calculation by treatment planning systems is approximately 50% where the region of isodose is less than 10% of the prescription dose [35, 36]. Therefore, the dose uncertainty in DVHs of the out-of-field organs might not be small. In our measurements, standard deviations in dose to the small organs such as thyroid and gonad, where detectors were located close to each other, were 6.2% and 4.9%, respectively. In larger organs, the uncertainty increased with larger distance between the detectors.

Even though the risk of radiogenic cancer is generally proportional to the absorbed dose, there are non-negligible uncertainties in the risk model including dose-response relationship for carcinogenesis and modeling parameters. The latest report on radiation risk suggested that decisive choices among the several dose-response models are not possible based on the empirical data [32]. Therefore, there may be large inherent uncertainties in the risk estimation.

An increase in LAR is attributed to the increase in leakage as a result of increased MUs from a traditional Linac. However, with the TOMO plan, the MUs are much greater than those of other modalities, yet the summed LAR was the lowest among modalities. Because while X-ray source of traditional Linac rotates around a patient continuously or at multiple static angles while the patient is lying still in a stationary couch, the TomoHDA system delivers the beam in a slice-by-slice fashion and each slice is collimated by the jaw in a moving couch, the definition of MU differs inherently for this modality. Therefore, MUs of TOMO cannot be explicitly compared to MUs of a traditional Linac.

The low summed LAR of the TOMO plan is mainly the result of two to three tangential IMRT fields delivered by a narrow field (5.0 cm). Therefore, our result might be limited to whole-breast radiation therapy. However, others have reported that the TomoHDA system provides better shielding [35, 37] and sharper dose gradient due to shorter source-to-skin distance (85 cm) and the narrower field width than those of the traditional Linac. These factors might have helped to further reduce the systemic LAR.

## CONCLUSIONS

This comparative study shows that even though an IMRT plan provides overall better plan quality and the lowest probability of pneumonitis, it causes the second highest total LAR, after a VMAT plan. Our results indicate that a TOMO plan provides a plan quality comparable to an IMRT plan and poses the lowest risk of LAR to in-field organs such as the ipsilateral lung, and to out-of-field organs. Therefore, TOMO can be a better treatment modality for younger patients who have a longer life expectancy.
